# Factors Associated with Para-Aortic Lymph Node Metastasis in High-Risk Endometrial Cancer

**DOI:** 10.3390/medicina61122189

**Published:** 2025-12-10

**Authors:** Fatma Ceren Güner, Elif Iltar, Müge Ateş Tıkız, Selen Doğan, Nasuh Utku Doğan, Hasan Aykut Tuncer, Tayup Şimşek

**Affiliations:** 1 Clinic of Gynecologic Oncology, Isparta City Hospital, Ministry of Health, 32200 Isparta, Turkey; 2 Division of Gynecologic Oncology, Department of Gynecology and Obstetrics, Akdeniz University, 07070 Antalya, Turkey; 3 Private Dogan Gynecology Clinic, 07050 Antalya, Turkey

**Keywords:** high-risk endometrial cancer, endometrial neoplasms, para-aortic lymph nodes, pelvic lymph nodes, lymphatic metastasis

## Abstract

*Background and Objectives*: Para-aortic lymph node involvement is a key prognostic factor in high-risk endometrial cancer. This study aimed to identify factors associated with para-aortic lymph node metastasis and to assess their predictive value for surgical decision-making. *Materials and Methods*: A retrospective analysis was conducted on 81 patients with high-risk endometrial cancer who underwent systematic pelvic and para-aortic lymphadenectomy between January 2015 and December 2024. Factors evaluated included histologic subtype, lymphovascular space invasion (LVSI), cervical stromal involvement, depth of myometrial invasion, and tumor diameter. Univariate and multivariate logistic regression analyses were performed to identify independent predictors of para-aortic metastasis. Receiver operating characteristic (ROC) analysis was used to determine the optimal tumor size threshold. *Results*: Para-aortic lymph node metastasis was identified in 21.0% of patients, and isolated para-aortic metastasis was observed in 2.5%. In univariate analysis, pelvic lymph node positivity, LVSI, cervical stromal invasion, deep myometrial invasion, and tumor size ≥ 3.55 cm were significantly associated with para-aortic spread. Multivariate analysis revealed that pelvic lymph node positivity was the only independent predictor (OR 39.0; 95% CI 5.06–301.46; *p* < 0.001). *Conclusions*: Pelvic lymph node status serves as a strong and independent predictor of para-aortic metastasis in high-risk endometrial cancer. A tumor diameter greater than 3.5 cm may also indicate an increased risk of para-aortic spread. These findings suggest that selective and individualized para-aortic assessment strategies may be considered to improve staging accuracy and optimize surgical planning in this patient population.

## 1. Introduction

Endometrial cancer represents the most frequently diagnosed gynecologic malignancy in high-income countries [1]. Although the majority of patients are diagnosed at an early stage and achieve favorable outcomes with surgery alone, those with high-risk histologic subtypes such as serous, clear cell, dedifferentiated, or mixed histologies, or with advanced-stage disease, are at substantially higher risk of lymph node metastasis. This risk adversely affects survival and often necessitates more aggressive adjuvant treatment strategies [2].

Sentinel lymph node biopsy (SLNB) has gained widespread acceptance for pelvic nodal assessment in low-risk endometrial cancer, supported by studies demonstrating its high diagnostic accuracy, sensitivity, and negative predictive value [3,4]. Recent prospective data, including the FIRES trial, have further supported the reliability of SLNB across diverse tumor characteristics and have contributed to its expanding use in selected high-risk histologic subtypes [4,5].

However, several studies have demonstrated that isolated para-aortic metastases may occur even in patients with negative pelvic sentinel lymph nodes, indicating a potential risk of understaging when para-aortic evaluation is omitted [6,7]. In a large multicenter retrospective study, Li et al. reported isolated para-aortic metastasis in 1.9% of patients with negative pelvic nodes, highlighting the importance of assessing the para-aortic region in selected cases [7]. In contrast, pelvic lymph node positivity has consistently been shown to strongly correlate with para-aortic lymph node involvement [8,9].

In line with these findings, the ESGO/ESTRO/ESP 2021 guidelines state that when systematic lymphadenectomy is performed in high-risk patients, both pelvic and para-aortic lymph node dissection should be carried out, while sentinel lymph node biopsy may be considered as an alternative approach [10]. Moreover, the revised 2023 FIGO staging system emphasizes the growing importance of tumor biology and histologic aggressiveness in determining staging and treatment decisions [11].

Despite these advances, no clear consensus exists regarding which factors most accurately predict para-aortic involvement in high-risk endometrial cancer, or under what circumstances systematic para-aortic dissection is truly warranted. Given the risks of both understaging and unnecessary surgical morbidity, this study aimed to identify factors associated with para-aortic lymph node metastasis in high-risk endometrial cancer.

## 2. Materials and Methods

**Figure 1:**
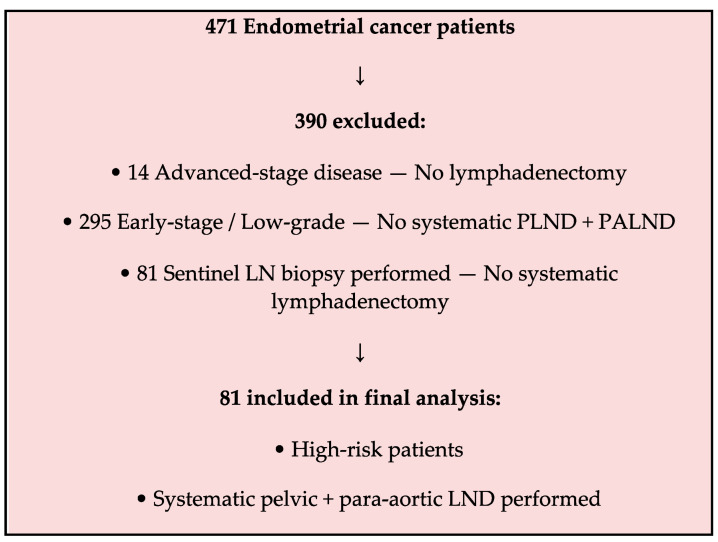
Flow diagram illustrating the patient selection process and exclusion criteria.

This retrospective study was conducted at a tertiary gynecologic oncology center between January 2015 and December 2024. Patients diagnosed with endometrial cancer who underwent surgical staging with systematic pelvic and para-aortic lymphadenectomy were included. Only those classified as high-risk according to the ESGO/ESTRO/ESP 2021 guidelines [10] were eligible for analysis. High-risk status was defined based on histopathologic criteria, including serous, clear cell, dedifferentiated, or mixed histologies, as well as FIGO grade 3 endometrioid carcinomas with deep myometrial invasion and/or lymphovascular space invasion (LVSI), and cervical stromal involvement. A detailed flow diagram of the patient selection process is presented in Figure 1.

Patients with missing data, inadequate lymph node sampling, or those who received neoadjuvant therapy were excluded.

All surgeries were performed by experienced gynecologic oncologists. Para-aortic lymphadenectomy was carried out in accordance with standardized institutional protocols, extending up to the level of the left renal vein. Clinical and pathological data—including age, body mass index (BMI), tumor size, depth of myometrial invasion, cervical stromal involvement, LVSI, and lymph node status—were retrieved from the hospital’s electronic medical records and pathology reports.

All statistical analyses were conducted using IBM SPSS Statistics for Windows, Version 23.0 (IBM Corp., Armonk, NY, USA). The normality of continuous variables was assessed using the Shapiro–Wilk test, Q–Q plots, and skewness–kurtosis values. Normally distributed variables were compared using the independent samples *t*-test, whereas non-normally distributed variables were analyzed using the Mann–Whitney U test. Categorical variables were compared using Pearson’s chi-square test or Fisher’s exact test, as appropriate. Receiver operating characteristic (ROC) curve analyses were performed to evaluate the discriminative ability of tumor size and the number of positive pelvic lymph nodes for predicting para-aortic metastasis. The performance of each ROC model was assessed using the area under the curve (AUC) with corresponding 95% confidence intervals.

To identify independent predictors of para-aortic metastasis, variables found to be significant in univariate analyses were included in a multivariate logistic regression model. Model fit was evaluated using the Hosmer–Lemeshow goodness-of-fit test, and explanatory power was reported using the Nagelkerke R^2^ statistic. A post hoc power analysis was conducted using G*Power version 3.1 (Heinrich Heine University, Düsseldorf, Germany) to assess the strength of the association between pelvic lymph node positivity and para-aortic metastasis, based on the cohort stratified by the ROC-derived cut-off [12].

This study was approved by the Clinical Research Ethics Committee of Akdeniz University (Approval No: TBAEK-534) and was conducted in accordance with the principles outlined in the Declaration of Helsinki.

## 3. Results

A total of 81 patients met the inclusion criteria and were analyzed. The mean age was 63.5 ± 12.3 years (range: 32–87), and the mean BMI was 31.2 ± 6.4 kg/m^2^ (range: 19.1–57.5). The most common histologic subtypes were mixed carcinoma (32.1%), serous carcinoma (25.9%), and clear cell carcinoma (12.3%). Other histologies included undifferentiated carcinoma (13.6%), carcinosarcoma (8.6%), and grade 3 endometrioid adenocarcinoma (7.4%), as shown in Table 1.

Para-aortic lymph node metastasis was identified in 17 patients, corresponding to an overall rate of 21.0%. Of these, two patients (2.5%) had no evidence of pelvic lymph node involvement and were classified as having isolated para-aortic metastasis. Among the PALN-positive patients, the histologic distribution included carcinosarcoma (*n* = 2), serous carcinoma (*n* = 2), mixed histology (*n* = 6), dedifferentiated carcinoma (*n* = 4), endometrioid carcinoma (*n* = 1), and clear cell carcinoma (*n* = 2). Of the two patients with isolated para-aortic metastasis, one had clear cell carcinoma and one had mixed histology.

Univariate analysis revealed significant associations between para-aortic metastasis and several factors. The presence of ≥1 positive pelvic lymph node was strongly associated with para-aortic spread (65.2% vs. 14.8%, *p* < 0.001). In addition, LVSI (*p* = 0.005), cervical stromal invasion (*p* = 0.008), deep myometrial invasion (≥50%) (*p* = 0.030), and tumor size ≥ 3.55 cm (*p* = 0.030) were all significantly associated with para-aortic involvement, as summarized in Table 2.

ROC curve analysis identified 3.55 cm as the optimal tumor size threshold for predicting para-aortic metastasis. This cutoff yielded an area under the curve (AUC) of 0.678 (95% CI: 0.54–0.81), with a sensitivity of 88.2% and specificity of 53.7%.

In the multivariate logistic regression analysis, only pelvic lymph node positivity remained a statistically significant independent predictor of para-aortic metastasis (OR: 39.0; 95% CI: 5.06 to 301.46; *p* < 0.001). Other variables, including tumor size ≥ 3.55 cm (OR: 4.32; 95% CI: 0.73 to 25.74), cervical stromal invasion (OR: 0.92; 95% CI: 0.16 to 5.15), lymphovascular space invasion (OR: 0.94; 95% CI: 0.15 to 6.11), and deep myometrial invasion (OR: 4.35; 95% CI: 0.70 to 27.06), did not reach statistical significance in the adjusted model, as shown in Table 3.

**Figure 2:**
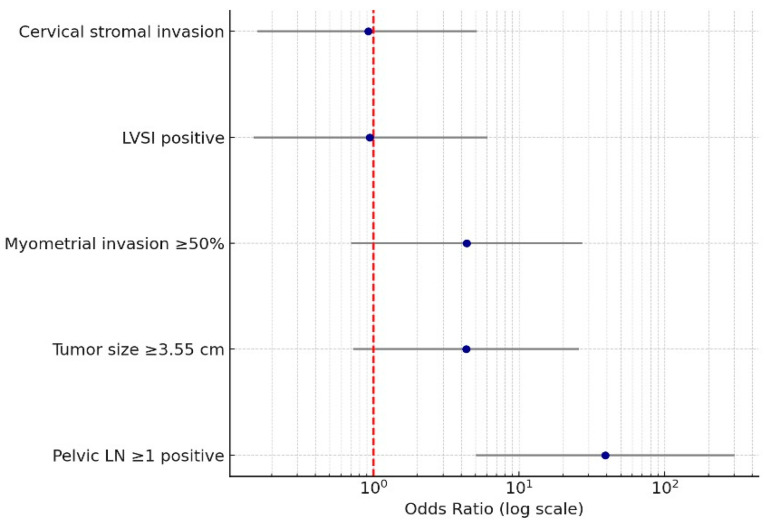
Factors Associated with Para-Aortic Lymph Node Metastasis. Forest plot of multivariate logistic regression analysis illustrating independent predictors of para-aortic lymph node metastasis. Odds ratios with 95% confidence intervals are presented on a logarithmic scale. The plot was generated using Python (version 3.12) in Google Colab, based on the regression output, with each covariate shown alongside its estimated effect size and confidence interval. The red dashed vertical line indicates the null effect line (odds ratio = 1).

The model showed a good overall fit, with a Nagelkerke R^2^ value of 0.601 and a non-significant Hosmer–Lemeshow test (*p* = 0.598), indicating adequate calibration. The overall correct classification rate was 82.0%. Results of the multivariate analysis are visually summarized in a forest plot (Figure 2), with a logarithmic *x*-axis used to accommodate the wide confidence intervals, particularly for pelvic lymph node positivity.

A post hoc power analysis was performed to evaluate the adequacy of the sample size for the association between pelvic lymph node positivity and para-aortic metastasis. The analysis yielded a power of 1.000 (α = 0.05, *n* = 81, OR = 39.0), confirming that the sample size was sufficient and the association was statistically robust.

## 4. Discussion

In this study, we aimed to identify factors associated with para-aortic lymph node metastasis in high-risk endometrial cancer. The rate of para-aortic metastasis in our cohort was 21%, which is comparable to previously reported data. In the SEPAL study published by Todo et al., a para-aortic lymph node metastasis rate of 29% was observed among high-risk patients who underwent both pelvic and para-aortic lymphadenectomy [13]. These findings underscore the potential importance of carefully evaluating the upper abdominal nodal region during surgical staging.

In our analysis, pelvic lymph node (LN) involvement emerged as the sole independent factor associated with para-aortic metastasis. This finding is consistent with the meta-analysis conducted by Han et al., which identified myometrial invasion (OR: 5.18), LVSI (OR: 3.46), and non-endometrioid histologic subtype (OR: 2.39) as significant risk factors for para-aortic lymph node metastasis, while pelvic LN metastasis showed the strongest association (OR: 16.72; 95% CI: 10.03–27.86) [14]. Similarly, Solmaz et al. reported that in multivariate analysis, both pelvic LN positivity and LVSI were independently associated with para-aortic dissemination [9]. Altay et al. further demonstrated that the presence of two or more metastatic pelvic lymph nodes significantly increased the risk of para-aortic spread, and in their multivariate model, pelvic LN status remained the only significant variable [15]. In a preoperative predictive model developed by Ueno et al., radiologically enlarged pelvic lymph nodes were found to be significantly associated with para-aortic metastasis and were among the strongest predictors included in the model [8]. Taken together, these findings highlight the critical importance of thorough and systematic evaluation of the para-aortic region in patients with pelvic nodal involvement, in order to avoid understaging and to ensure optimal treatment planning.

The association between tumor size and lymph node metastasis in endometrial cancer has been consistently demonstrated in the literature. Studies have shown that a tumor diameter of ≥2 cm is significantly correlated with higher rates of pelvic lymph node involvement [16]. Tumor size is also a key component of the widely accepted Mayo criteria, which identify patients with grade 1–2 endometrioid tumors, no deep myometrial invasion, and a tumor diameter less than 2 cm as having a very low risk of nodal metastasis [17]. Similarly, in a retrospective analysis by Zhang et al., tumor size greater than 2 cm, along with deep myometrial invasion and high histologic grade, was significantly associated with lymph node metastasis, and these factors remained independent predictors in multivariate analysis [18]. In our study, ROC curve analysis identified 3.55 cm as the optimal tumor size threshold for predicting para-aortic lymph node metastasis. This cutoff demonstrated moderate discriminative performance, with a sensitivity of 88.2% and specificity of 53.7% (AUC: 0.678; 95% CI: 0.54–0.81). Although tumor size was a significant predictor in univariate analysis, it did not retain statistical significance in the multivariate logistic regression model (OR: 4.32; 95% CI: 0.73–25.74; *p* > 0.05). Nevertheless, a rounded threshold of 3.5 cm may serve as a clinically useful reference point. Tumors exceeding this size may warrant more careful evaluation in terms of para-aortic metastatic risk.

In our cohort, the rate of isolated para-aortic lymph node metastasis was found to be 2.5%. A similar rate of 2.4% was reported in a retrospective study, while a meta-analysis estimated this incidence at 2.58% [14,19]. In another series, this rate was reported as 4% [15]. Although rare, isolated para-aortic metastasis should still be considered during surgical staging, particularly in patients with high-risk endometrial cancer. In a recent study by Benseler et al., 5.8% of high-grade endometrial cancer patients with negative pelvic sentinel lymph nodes were found to have isolated para-aortic metastases, all of whom had serous or carcinosarcoma histology and outer half myometrial invasion [6]. Consistent with our findings, Lai et al. demonstrated that the survival benefit of para-aortic lymphadenectomy was limited to specific subgroups of high-grade endometrial cancer, particularly those with grade 3 endometrioid histology and lymphovascular space invasion [20]. According to Fotopoulou et al., 3.1% of patients had isolated para-aortic lymph node metastases, with some located above the level of the inferior mesenteric artery. Importantly, their multivariate analysis revealed that lymph node involvement was the sole independent predictor of overall survival [21]. These findings collectively support a selective, risk-adapted approach to para-aortic assessment in high-risk endometrial cancer.

The potential therapeutic value of para-aortic lymphadenectomy has also been emphasized in previous studies. The SEPAL study demonstrated that adding para-aortic lymphadenectomy to pelvic lymphadenectomy was associated with improved survival in patients with intermediate- and high-risk endometrial cancer, and that this benefit persisted independently of adjuvant chemotherapy or radiotherapy [13]. Therefore, evaluation of the para-aortic region represents a critical step in the surgical management of high-risk patients.

To support surgical decision-making regarding the necessity of para-aortic lymphadenectomy, several clinical models have been developed to identify patients at high risk of lymph node metastasis in the preoperative setting. Among the most notable is the model proposed by Ueno et al., which combines 10 clinical variables to predict lymph node metastasis in patients with endometrial cancer with high accuracy [8]. This model incorporates classical prognostic factors such as CA125 level, tumor volume, depth of myometrial invasion, and histological subtype, along with radiologic evidence of para-aortic lymph node enlargement, which was identified as a significant predictor [8]. In contrast, our multivariable analysis revealed that pelvic lymph node positivity was the only independent predictor of para-aortic metastasis. Notably, the presence of para-aortic metastases in some patients with negative pelvic nodes suggests that conventional risk factors alone may not be sufficient for guiding surgical decisions. In such cases, especially when sentinel lymph node biopsy is not feasible or proves unsuccessful, individualized approaches that integrate radiologic findings, tumor biology, and clinical parameters may offer more reliable guidance in determining the extent of surgical intervention.

In a large-scale national database study involving patients with non-endometrioid endometrial cancer, Venigalla et al. reported that the addition of para-aortic lymphadenectomy to pelvic lymphadenectomy significantly improved survival, particularly in the serous carcinoma subgroup (HR: 0.85; 95% CI: 0.79–0.91) [22]. In our study, which excluded low-risk patients, multivariable analysis identified pelvic lymph node positivity as the sole independent predictor of para-aortic metastasis. In this context, detailed preoperative imaging and intraoperative assessment may provide additional support when determining the need and extent of para-aortic dissection. Intraoperative frozen assessment of sentinel lymph nodes has been shown to provide rapid and reliable information that may assist surgeons in real-time decision-making during the primary operation [23]. The fact that pelvic lymph node positivity was the strongest indicator of para-aortic metastasis in our cohort underscores its critical role in individualizing surgical strategies for high-risk patients.

A recent meta-analysis further supports a selective, risk-adapted approach, demonstrating that combined pelvic and para-aortic lymphadenectomy is associated with improved overall survival, although recurrence rates do not appear to be significantly reduced [24]. However, this surgical strategy was also associated with an increased risk of postoperative complications and prolonged operative times [24]. The decision to perform para-aortic lymphadenectomy should therefore be individualized, balancing potential survival benefits against increased operative time and postoperative morbidity.

In recent years, molecular classification has gained increasing importance in the risk stratification and management of endometrial cancer. The ESGO/ESTRO/ESP 2021 guidelines define four molecular subtypes of endometrial carcinoma: POLE ultramutated, mismatch repair deficient (MMRd), p53-aberrant, and NSMP (no specific molecular profile) [10]. Each subtype is associated with distinct prognostic implications and patterns of lymphatic dissemination [25]. Notably, lymph node metastasis has been reported more frequently in tumors with p53-aberrant and MMRd profiles, which are also more commonly associated with advanced-stage disease and the need for adjuvant chemotherapy [26,27]. However, molecular profiling was not performed in our study, which limits our ability to evaluate the relationship between these molecular features and para-aortic lymph node metastasis.

Overall, this study suggests that systematic para-aortic lymphadenectomy in high-risk endometrial cancer may play a critical role not only in accurate staging but also in guiding adjuvant treatment decisions and potentially improving survival outcomes. Our findings underscore the value of identifying pelvic lymph node positivity as a strong predictor of para-aortic metastasis, while also highlighting the limitations of relying solely on classical risk factors or sentinel lymph node biopsy. Future prospective, multicenter studies that incorporate molecular profiling and advanced pathological evaluation techniques will be essential to refine predictive models and improve surgical planning in this patient population.

This study has several limitations. First, the relatively small number of patients with para-aortic lymph node metastasis reduced the statistical power of the multivariable analyses and contributed to the wide confidence interval observed for pelvic lymph node positivity. In addition, although the cohort included multiple high-grade histologic subtypes, the small number of cases within each category prevented meaningful subtype-specific analyses and limited our ability to fully evaluate the potential impact of histologic heterogeneity. The absence of molecular classification data represents another important limitation, as these molecular characteristics significantly influence patterns of spread and prognosis. Finally, the retrospective nature of the study introduces inherent constraints, including selection bias, variations in surgical practice over time, and incomplete clinical or pathological documentation.

## 5. Conclusions

Para-aortic lymph node metastasis was observed in 21% of patients with high-risk endometrial cancer, and pelvic lymph node positivity was identified as the only independent predictor. The presence of isolated para-aortic metastases suggests that limited pelvic staging may underestimate the true extent of disease. In addition, a tumor diameter greater than 3.5 cm was associated with high sensitivity for predicting para-aortic spread and may serve as a useful parameter in preoperative risk assessment. These findings suggest that individualized and selective para-aortic assessment strategies may be considered to improve staging accuracy and guide surgical planning in high-risk patients. The incorporation of molecular profiling and radiologic features into preoperative decision-making may further support personalized surgical approaches in this population.

## Figures and Tables

**Table 1:** Baseline Demographic and Clinical Characteristics of the Study Population.

Characteristics	Total (*n* = 81)
Age (years), mean ± SD	63.5 ± 12.3
BMI (kg/m^2^), mean ± SD	31.2 ± 6.4
Histologic subtype (*n*, %)	
Mixed	26 (32.1)
Serous	21 (25.9)
Clear cell	10 (12.3)
Undifferentiated	11 (13.6)
Carcinosarcoma	7 (8.6)
Grade 3 Endometrioid	6 (7.4)
LVSI positive (*n*, %)	29 (35.8)
Cervical stromal invasion (*n*, %)	23 (28.4)
Myometrial invasion ≥ 50% (*n*, %)	38 (46.9)
Tumor size ≥ 3.55 cm (*n*, %)	38 (46.9)

**Table 2:** Factors Associated with Para-Aortic Metastasis: Univariate Analysis.

Variable	Para-Aortic Metastasis (+) (*n* = 17)	Para-Aortic Metastasis (−) (*n* = 64)	*p*-Value
Pelvic LN ≥ 1 positive	15 (88.2%)	8 (12.5%)	<0.001
LVSI positive	13 (76.5%)	16 (25.0%)	0.005
Cervical stromal invasion	10 (58.8%)	13 (20.3%)	0.008
Myometrial invasion ≥ 50%	13 (76.5%)	25 (39.1%)	0.030
Tumor size ≥ 3.55 cm	13 (76.5%)	25 (39.1%)	0.030

**Table 3:** Multivariate Logistic Regression Analysis of Predictors of Para-Aortic Metastasis.

Variable	OR (95% CI)	*p*-Value
Pelvic LN ≥ 1 positive	39.0 (5.06–301.46)	<0.001
LVSI positive	0.94 (0.15–6.11)	0.95
Cervical stromal invasion	0.92 (0.16–5.15)	0.93
Myometrial invasion ≥ 50%	4.35 (0.70–27.06)	0.11
Tumor size ≥ 3.55 cm	4.32 (0.73–25.74)	0.11

## Data Availability

The data presented in this study are available on request from the corresponding authors due to privacy and ethical considerations.

## Abbreviations

The following abbreviations are used in this manuscript:

AUC	Area under the curve
BMI	Body mass index
CI	Confidence interval
ESGO/ESTRO/ESP	European Society of Gynaecological Oncology/European Society for Radiotherapy and Oncology/European Society of Pathology
FIGO	International Federation of Gynecology and Obstetrics
HR	Hazard ratio
LN	Lymph node
LVSI	Lymphovascular space invasion
MMRd	Mismatch repair deficient
NSMP	No specific molecular profile
OR	Odds ratio
POLE	Polymerase epsilon ultramutated subtype
ROC	Receiver operating characteristic
SLNB	Sentinel lymph node biopsy
